# The *CcMAK2* gene is required for proper asexual fruiting body formation in *Cordyceps cicadae*

**DOI:** 10.3897/imafungus.17.185776

**Published:** 2026-04-15

**Authors:** Lanya Chen, Xiaoli Tian, Liping Chen, Yanting Xiao, Jie Han, Jiehong Zhao

**Affiliations:** 1 College of Pharmacy, Guizhou University of Traditional Chinese Medicine, Guiyang, 550025, Guizhou, China College of Pharmacy, Guizhou University of Traditional Chinese Medicine Guiyang China; 2 Guizhou Key Laboratory of Microbio and Infectious Disease Prevention & Control, Guizhou University of Traditional Chinese Medicine, Guiyang, 550025, Guizhou, China Guizhou Key Laboratory of Microbio and Infectious Disease Prevention & Control, Guizhou University of Traditional Chinese Medicine Guiyang China; 3 Guizhou Key Laboratory of Modern Chinese Medicine Creation, Guizhou University of Traditional Chinese Medicine, Guiyang, 550025, Guizhou, China Guizhou Key Laboratory of Modern Chinese Medicine Creation, Guizhou University of Traditional Chinese Medicine Guiyang China

**Keywords:** *Cordyceps cicadae*, fruiting body, *MAK2* gene, MAPK pathway, ROS

## Abstract

*Cordyceps cicadae* is a traditional precious medicinal and edible entomogenous fungus. Its asexual fruiting bodies are highly valued in natural health food. Currently, the intrinsic mechanisms regulating fruiting body development in *C. cicadae* remain largely elusive. The Fus3/MAK2 homolog within the MAPK signaling pathway plays a crucial role in sexual reproduction and pathogenesis in filamentous fungi, yet its function in the fruiting body development of *C. cicadae* has not been reported. Therefore, this study focused on *CcMAK2* from *C. cicadae*, constructing gene overexpression and knockout strains to analyze its impact on fungal growth and development. The results demonstrated that the knockout strain (ΔCcMAK2) failed to penetrate the insect cuticle from inside, and completely lost the ability to form fruiting bodies. In contrast, the wild-type (WT) and overexpression strain (CcMAK2^OE^) developed normally, indicating that *CcMAK2* is essential for fruiting body formation in *C. cicadae*. Functional analysis revealed that the ΔCcMAK2 mutant exhibited significantly reduced levels of H_2_O_2_ and O_2_^.^–during the fruiting stage, accompanied by increased activities of CAT and GR enzymes, as well as elevated chitinase activity. These findings suggest that the deletion of *CcMAK2* is associated with alterations in ROS homeostasis and cell wall-related enzyme. Gene expression analysis further showed that the deletion of *CcMAK2* led to altered transcript levels of the downstream transcription factor *Ste12* and the cell wall integrity pathway-related gene *CcSO*. These results suggest that *CcMAK2* may be involved in regulating these genes, potentially contributing to its role in fruiting body development. This study provides foundational insights into the role of *CcMAK2* in fruiting body development in *C. cicadae* and lays groundwork for further mechanistic studies in *Cordyceps* and other fungi.

Graphical abstract

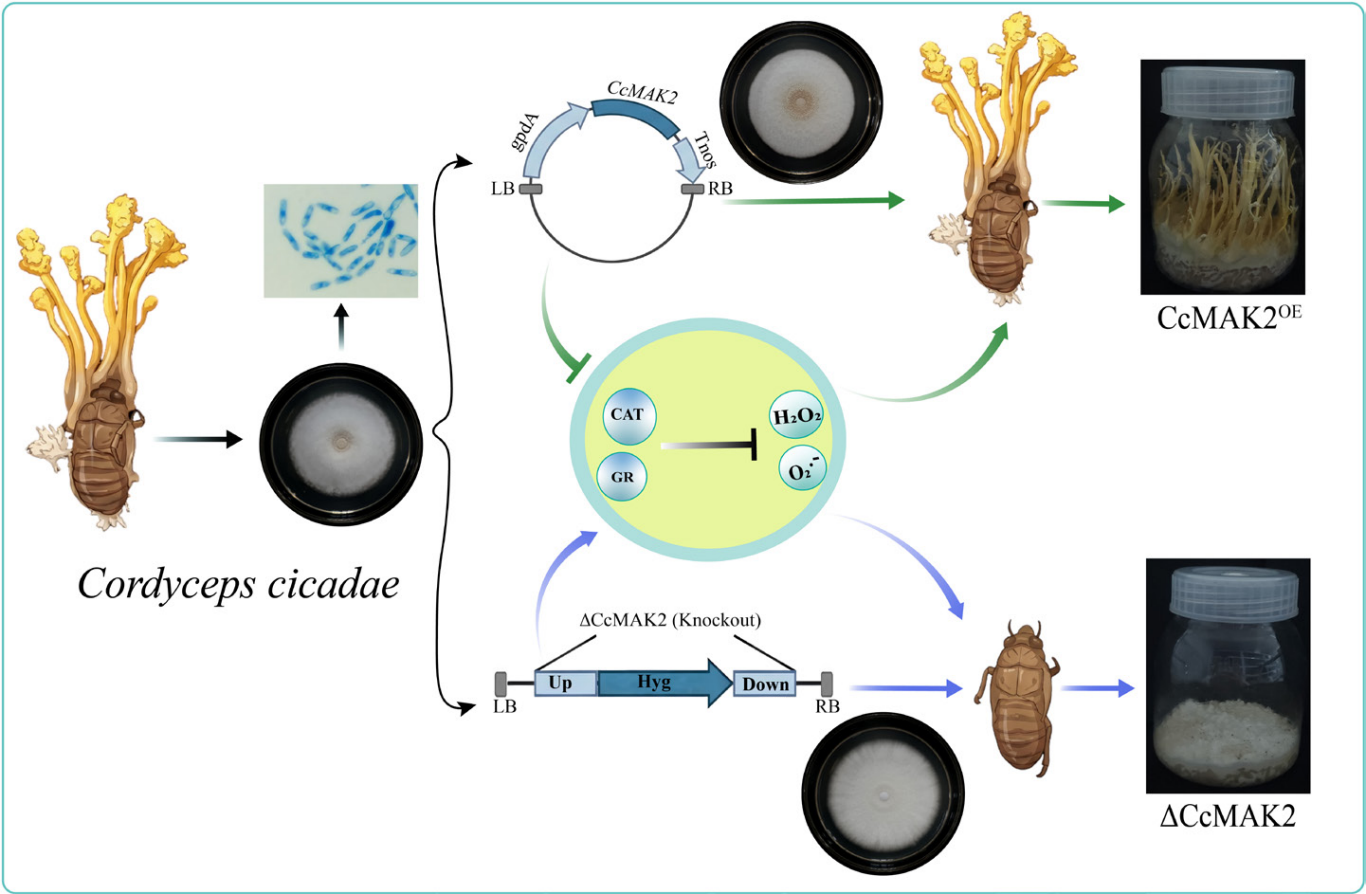

## Introduction

*Cordyceps cicadae* (=*Isaria cicadae*) (Kepler et al. 2017) is an entomopathogenic fungus that parasitizes cicada nymphs, and is widely distributed in China and other Asian countries. *Cordyceps cicadae* primarily completes its life cycle through asexual development, its sexual cycle is rarely observed. It possesses significant medicinal and nutritional value, and its fruiting bodies are highly valued in both traditional Chinese medicine and cuisine. Modern research has shown that *C. cicadae* is rich in various bioactive compounds, including adenosine (Zeng et al. 2014), polysaccharides (Wei et al. 2016), cordycepic acid (Shi et al. 2022), and N6-(2-hydroxyethyl)-adenosine (HEA) (Olatunji et al. 2016). These compounds contribute to a broad spectrum of pharmacological activities, such as antioxidant effects, renal function improvement, immunomodulation, anti-fatigue, anti- inflammatory, and anti-diabetic properties (Kim et al. 2012; Zhu et al. 2014; Wang et al. 2018; Wang et al. 2019). Notably, the pharmacological profile of *C. cicadae* is highly similar to that of *Ophiocordyceps sinensis* and *Cordyceps militaris*, coupled with its high biosafety, making it an ideal alternative resource to these two well-known cordycipitoid fungi (Nxumalo et al. 2020). In China, Korea, and Japan, *C. cicadae* has been used as a traditional natural health food (Takano et al. 1996), demonstrating significant potential for applications in the pharmaceutical, food, and cosmetic industries.

The fruiting body of *C. cicadae* lacks sexual sporulation structures but produces a large number of conidia at its apex. This unique asexual type developmental characteristic warrants in-depth investigation. In filamentous fungi, fruiting body development and differentiation are coordinately regulated by multiple highly conserved signaling pathways. Among these, the mitogen-activated protein kinase (MAPK) signaling cascade plays a central role. Key regulatory modules coordinating hyphal growth, morphogenesis, and fruiting body development include the pheromone response (PR) and cell wall integrity (CWI) pathways within the MAPK cascade, the nicotinamide adenine dinucleotide phosphate (NADPH) oxidase complexes (NOX1 and NOX2) that generate reactive oxygen species (ROS) signals, and the striatin-interacting phosphatase and kinase (STRIPAK) complex that regulates G-protein signaling and cell polarity. These modules collectively form an integrated regulatory network (Pöggeler et al. 2018; Riquelme et al. 2018; Fischer and Glass 2019).

Five canonical MAPK pathways have been identified in *Saccharomyces cerevisiae*, each implicated in distinct cellular processes (Hamel et al. 2012). Among these, Fus3-MAPK, a mitogen-activated protein kinase, is the core component of the PR pathway within the MAPK cascade, whose regulatory functions are extensive, encompassing key biological processes such as fungal cell growth, development, and pathogenicity, garnering significant research attention (Bayram et al. 2012; Lemos et al. 2018; Yang et al. 2020). In yeast, the specificity of this pathway is largely determined by the substrate selectivity of Fus3, which specifically phosphorylates the multifunctional protein Far1 (Breitkreutz et al. 2001). Phosphorylated Far1 arrests the cell cycle by inhibiting the activity of the Cdc28 cyclin-dependent kinase in G1 phase, preparing the cell for sexual development. Additionally, it interacts with the G protein βγ subunits to recruit polarity establishment proteins toward the pheromone gradient, thereby directing polarized cell growth (Breitkreutz et al. 2001; Muller et al. 2016). Simultaneously, Fus3 activates the transcription factor Ste12 by inhibiting the transcriptional repressors Dig1 and Dig2, which in turn initiates the transcription of a set of mating-specific genes, including Far1 and Fus1 (Pomeroy et al. 2021). The Fus1 protein is a key factor executing cell membrane fusion, and its expression level is frequently used as a reporter for the activity of this pathway (Gammie et al. 1998; Pujari and Cullen 2023). Therefore, Fus3 ensures the proper execution of the sexual developmental program through the precise regulation of Far1 and the Ste12-Fus1 axis.

In *Neurospora crassa*, the ortholog of Fus3-MAPK, MAK2, regulates conidiation, hyphal fusion, and sexual development (Pandey et al. 2004; Li et al. 2005). In *Aspergillus nidulans*, the Fus3-MAPK signaling module comprises Fus3, the upstream kinases Ste7 and Ste11, and the adaptor protein Ste50; however, only Fus3 can enter the nucleus where it phosphorylates the sexual development regulator Ste12 and the global regulator VeA. Ste12 mediates the regulation of developmental genes, particularly those contributing to sexual development, whereas VeA activation triggers the assembly of the velvet complex, which modulates both sexual development and secondary metabolism (Atoui et al. 2008; Schamber et al. 2010; Bayram et al. 2012). Deletion of *Fus3* in *Arthrobotrys oligospora* results in slowed growth, reduced sporulation, and aberrant hyphal and spore morphology (Xie et al. 2023). In *Beauveria bassiana*, the Ste12-like protein (*BbSte12*) and the Fus3/Kss1 MAPK homolog (*Bbmpk1*) interact within the nucleus, and the phosphorylation of *BbSte12* by *Bbmpk1* is essential for the penetration of the insect cuticle by *B. bassiana*. This pathway is also involved in other processes controlling conidiation, growth, hyphal differentiation, and the oxidative stress response (Zhao et al. 2023). In *Magnaporthe grisea*, *Pmk1* (a homolog of *Fus3*) regulates appressorium formation and is required for host cuticle penetration and invasive growth (Xu and Hamer 1996). In *Aspergillus nidulans*, *MpkB* (a homolog of *Fus3*) is involved in hyphal growth, conidiation, conidial viability, sexual development, and autolysis (Kang et al. 2013). In *Pyrenophora teres* (Ruiz-Roldán et al. 2001) and *Cochliobolus heterostrophus* (Lev et al. 1999), Fus3/Kss1-type MAPK genes affect conidiation. These findings collectively demonstrate that Fus3/MAK2 plays a crucial role in fungal vegetative or sexual cell fusion, secondary metabolism, and pathogenicity.

Fruiting body development is a critical process in macrofungi, and its formation involves complex genetic mechanisms (Wang et al. 2025). Genes associated with the MAPK signaling pathway have been demonstrated to play roles in the sexual fruiting body development of various fungi, including *C. militaris* (Zheng et al. 2011) and *Coprinopsis cinerea* (Zhao et al. 2022). Although studies directly addressing the regulatory role of Fus3/MAK2 in fruiting body development are relatively scarce, *MAK2*, as a key component of the MAPK pathway, likely regulates processes such as cell differentiation, morphogenesis, and secondary metabolism through analogous mechanisms during fruiting body development in filamentous fungi (Bayram et al. 2012). Therefore, systematically dissecting the function of *MAK2* in *C. cicadae* will not only reveal the regulatory mechanisms of its asexual fruiting body development from a key signaling node but also provide novel insights into the developmental biology of entomopathogenic fungi. In this study, by constructing *MAK2* knockout and overexpression strains in *C. cicadae*, we systematically analyzed the role of *MAK2* in regulating mycelial growth, sporulation, and asexual fruiting body development.

## Materials and methods

### Fungal strain and culture media

The wild-type (WT) strain of *C. cicadae* (=*I. cicadae*) was generously provided by the Institute of Fungal Resources, Guizhou University, and was originally isolated from Huaxi District, Guiyang City, Guizhou Province, China. A pure culture of this strain has been deposited in the China Center for Type Culture Collection (CCTCC) under the accession number CCTCC NF 20081502. Fungal colonies were cultivated on Potato Dextrose Agar (PDA, purchased from Beijing Solarbio Science & Technology Co., Ltd.) or Sabouraud Dextrose Agar (SDA, composed of glucose 40 g/L, peptone 10 g/L, and agar 15 g/L) plates. For fruiting body induction, a solid rice-based medium was used, per litre consisting of 20 g rice, 1 g egg white, and 30 mL nutrient solution. The nutrient solution contained: 3.6 g glucose, 3.6 g silkworm pupa powder, 0.2 g peptone, 0.2 g KH_2_PO_4_, 0.4 g MgSO_4_, and 0.4 g citric acid, dissolved in 1000 mL distilled water. The compositions of the rice solid medium, the induction medium (IM) for *Agrobacterium tumefaciens*-mediated transformation, and the co-cultivation medium are described in Chen et al. (2025).

### Identification and sequence analysis of the *CcMAK2* gene in *C. cicadae*

Based on the *MAK2* gene sequence (FungiDB: NCU02393) from *N. crassa*, a BLAST analysis was performed against the *C. cicadae* genome MWMN01000005.1 (Lu et al. 2017) in the NCBI database to identify homologous sequences. Primers were designed using Primer 3 (all primers used are listed in Table 1) for the obtained *MAK2* sequence, followed by cloning and sequencing. The resulting gene sequence has been deposited in GenBase (https://ngdc.cncb.ac.cn/genbase/) under the accession number C_AA136328.1. To elucidate the phylogenetic relationship of CcMAK2 and its homologs, MAK2 homologous protein sequences from 14 representative species, including cordycipitoid fungi and other reported ascomycetes, were retrieved for phylogenetic analysis and domain prediction to determine the evolutionary position of CcMAK2. Multiple sequence alignment was conducted using MEGA11, and a phylogenetic tree was constructed using the neighbor-joining method (with bootstrap replicates set to 1000). Protein domain analysis was performed using the InterPro database (https://www.ebi.ac.uk/interpro/), and visualization was carried out using the online tool IBS 2.0 (https://ibs.renlab.org/).

**Table 1.** Primer sequences.

Primer name	Primer sequence (5’→3’)	Purpose
MAK2-F	CAAACCCTCCCAATAACGCC	Full-length amplification
MAK2-R	AACCAACTGCACTTCTCCCT	
MAK2-ck-F	CAAACCCTCCCAATAACGCC	Knockout strain verification
MAK2-ck-R	AACCAACTGCACTTCTCCCT	
Hyg-F	GTGCTTGACATTGGGGAGTT	Transformant confirmation
Hyg-R	GATGTTGGCGACCTCGTATT	
mCherry-F	GCCCCGTAATGCAGAAGAAG	Overexpression strain verification
mCherry-R	GTGTAGTCCTCGTTGTGGGA	
q-MAK2-F	GACATGCACCGAGTTATCCG	*MAK2* RT-qPCR
q-MAK2-F	GGAAGGCTTGAGGTCTCTGT	
q-CcSO-F	GAAATCCACTCATGTCGCCC	*CcSO* RT-qPCR
q-CcSO-R	AGTTGGAGCTTGCGAAACAG	
q-Ste12-F	CTCGTATGGGAGCAACAACG	*Ste12* RT-qPCR
q-Ste12-R	ACCAACAGAGACGGAGACAG	
q-Fus1-F	CATCGTGTCCAGCTCGACTT	*Fus1* RT-qPCR
q-Fus1-R	TTGCTGAGAGCGATCGAGAC	
q-Far1-F	CTGCCTCAAGATGCTTGGGA	*Far1* RT-qPCR
q-Far1-R	AGAATGTCGCCCCAACTCAG	
q-Vea-F	TCGCCATCTTATCCGGAGTC	*Vea* RT-qPCR
q-Vea-R	TGGTTCGTAGGCATCCAGTT	
GAPDH-F	GGTGCCAAGAAGGTCATCAT	Reference gene *GAPDH* RT-qPCR
GAPDH-R	GCGGTGTAGGAGTGAATGGT	

### Construction of gene overexpression and knockout vectors

The full-length *CcMAK2* gene was amplified using primers MAK2-oe-F/R, which contained homology arms to the vector. Subsequently, the *CcMAK2* gene was ligated with the linearized backbone vector pCAMBIA1303-mCherry using the EasyGeno One-Fragment Recombinant Cloning Kit (Tiangen Biotech, China), resulting in the construction of the *CcMAK2* overexpression vector pCAMBIA1303-MAK2-mCherry for fungal transformation. The knockout vector was constructed via a homologous recombination strategy. The Up800-Hyg-Down800 fragment, containing 800 bp homologous arms from the upstream and downstream regions of the *CcMAK2* gene, was ligated into a linearized backbone vector using the aforementioned method, yielding the *CcMAK2* knockout vector pDHtsk-Hyg-MAK2. The resulting vectors were then transformed into *Agrobacterium tumefaciens* GV3101 competent cells using the freeze-thaw method. The transformed cells were plated on LB agar plates containing 50 mg/L kanamycin (Kan) to select for resistant colonies. Positive *Agrobacterium* strains were verified by PCR analysis.

### Genetic transformation of *C. cicadae*

The fungal colonies grown on PDA plates were rinsed with sterile water, and the collected and filtered conidia of *C. cicadae* were suspended in 0.05% Tween 80, adjusted to a concentration of 1 × 10^5^ conidia/mL. The confirmed positive *A. tumefaciens* cells were cultured in liquid LB medium containing 50 mg/L Kan for activation. After centrifugation and supernatant removal, the bacterial pellets were resuspended in IM medium to an OD_600_ of 0.15–0.20 and incubated under the same conditions for 4–6 h until the OD_600_ reached 0.60–0.80. A mixture of 100 μL of the fungal spore suspension and 100 μL of *A. tumefaciens* culture was spread onto modified CO-IM plates overlaid with a 0.45 μm nitrocellulose membrane, followed by co-cultivation at 25 °C for 3–5 days. The nitrocellulose membrane was then transferred onto SDA medium supplemented with 200 mg/L hygromycin and 300 mg/L cefotaxime, and incubated at 25 °C for 3–6 days. Putative transformants were screened by PCR using the primer pairs MAK2-ck-F/R, Hyg-F/R, and mCherry-F/R. A total of 7 *CcMAK2* knockout transformants and 5 *CcMAK2* overexpression transformants were obtained. For all subsequent quantitative phenotypic analyses, three randomly selected and verified independent transformants for each construct were used as biological replicates, and the results are presented as mean ± SD. The images of colony plates presented in the figures are representative.

### Phenotypic observation of various strains

The overexpression strain (CcMAK2^OE^), knockout strain (ΔCcMAK2), and wild-type strain (WT) were separately inoculated on PDA plates and cultured at 25 °C in darkness for 7 days. Conidia were then harvested and diluted to a concentration of 1 × 10^6^ conidia/mL using 0.05% Tween-80. A 1.5 μL aliquot of the spore suspension was inoculated onto both PDA and SDA plates and cultured at 25 °C for 14 days. The colony diameter was measured daily, and the growth index was calculated. To determine conidial yield, 1.5 μL of the spore suspension was inoculated onto PDA and SDA plates and cultured at 25 °C. At 7 and 14 days of cultivation, conidia were harvested by rinsing the plates with 20 mL of 0.05% Tween-80. The suspension was filtered through three layers of lens cleaning paper, and the conidial yield was quantified using a hemocytometer. Furthermore, mycelia were stained with lactophenol cotton blue stain for microscopic observation of the conidiogenous structures.

### Virulence assay of different fungal strains against *Galleria mellonella*

To assess the impact of the *CcMAK2* gene on the virulence of *C. cicadae* conidia against larvae of the greater wax moth, *Galleria mellonella*, a bioassay was performed. Larvae were immersed for 10 seconds in a conidial suspension (1 × 10^7^ conidia/mL) of the respective fungal strain (Guan et al. 2024). Mortality was recorded at 12-hour intervals starting 24 hours post-inoculation until all larvae had died. Each treatment consisted of three biological replicates with 30 larvae per replicate, and the median lethal time (LT_50_) was calculated as the virulence index. Larvae that died at the same time point were transferred to new Petri dishes and maintained under humid conditions at 25 °C to observe the process of hyphal growth emerging from the insect cuticle.

### Artificial cultivation of *C. cicadae* fruiting bodies

To assess the fruiting body formation capacity of different strains, CcMAK2^OE^, ΔCcMAK2, and WT were inoculated into potato dextrose broth (PDB) and cultured at 25 °C with shaking at 150 rpm for 5 days. Subsequently, 1 mL of the fermentation broth was transferred to a rice-based solid medium and cultivated for 30 days. The number and height of the fruiting bodies were then recorded. The biological efficiency (BE) for fruiting body formation was calculated as follows: BE (%) = (Fresh weight of fruiting bodies / Dry weight of the substrate) × 100%. All experiments were performed with three replicates per group.

### Determination of H_2_O_2_, O_2_^.^–, antioxidant enzyme, and chitinase activities

Fruiting bodies (0.1 g) were collected, and the assays were performed following the manufacturer’s protocols of the respective commercial kits for H_2_O_2_, O_2_^.^–, catalase (CAT), superoxide dismutase (SOD), glutathione reductase (GR), and chitinase. All kits were purchased from Solarbio Science & Technology Co., Ltd. (Beijing, China).

### Quantitative real-time PCR (RT-qPCR) analysis

Total RNA was extracted from mycelial and fruiting body stages of different strains cultured on rice medium. Reverse transcription was performed using the FastKing gDNA Dispelling RT SuperMix kit (Tiangen Biotech). Quantitative PCR was conducted on an ABI StepOne system (Applied Biosystems, USA) using the SYBR Green method to analyze the expression levels of *CcMAK2*, *Ste12* (GenBank: NM_001179214.1), *Fus1* (GenBank: NM_001178672.1), *Far1* (GenBank: NM_001181590.1), *CcSO* (GenBank: C_AA113794.1), and *Vea* (GenBank: XM_747526.2). The *GAPDH* gene (GenBank: XM_018846033.1) was used as the internal reference. Gene expression levels were calculated using the 2^−ΔΔct^ method.

### Statistical analysis

Experimental data were analyzed by one-way analysis of variance (ANOVA) using GraphPad Prism software, and bar graphs were plotted. A p-value of less than 0.05 was considered statistically significant.

## Results

### Sequence analysis of *CcMAK2* in *C. cicadae*

**Figure 1.**
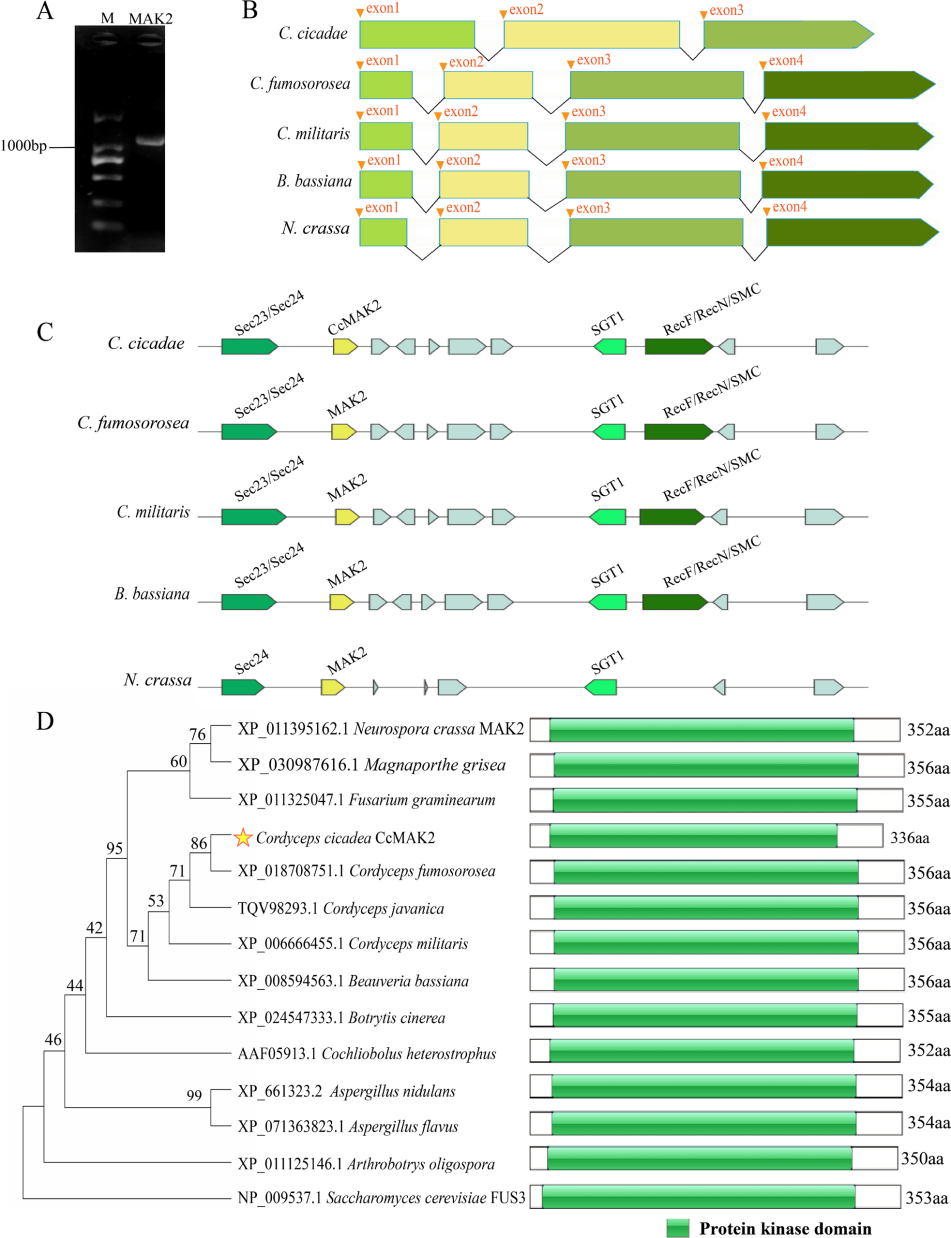
Cloning and sequence analysis of *CcMAK2* in *C. cicadae*. **A** Cloning of *CcMAK2* from *C. cicadae*; **B** Comparative analysis of the *MAK2* gene structure across diverse ascomycetes. Exons are represented by rectangular boxes, and introns are indicated by bent lines; **C** Genomic localization characteristics of the *MAK2* gene in various ascomycetes; **D** Phylogenetic relationship and conserved domain comparison of the MAK2 amino acid sequences from different ascomycetes. M: DNA marker 2000. aa: amino acids.

To clone the *CcMAK2* gene from *C. cicadae*, a homologous sequence was first identified by performing a BLAST search against the *C. cicadae* genome database in NCBI, using the *MAK2* sequence (FungiDB: NCU02393) from *N. crassa* as a query. Specific primers were then designed based on this homologous sequence to amplify the target gene from the genomic DNA of the *C. cicadae* DJS strain (Fig. 1A). Sequencing results confirmed that the cloned fragment was consistent with the intended target, and it was consequently designated as *CcMAK2*. The *CcMAK2* gene comprises 1011 bp, encoding a protein of 336 amino acids with a predicted molecular weight of approximately 39.1 kDa.

To investigate the evolutionary characteristics of the *C. cicadae* gene *CcMAK2*, its gene structure was analyzed. The full-length *CcMAK2* gene comprises three exons and two introns. To assess the conservation of this gene structure, homologous genes from other entomopathogenic and model fungi were compared, including *Cordyceps fumosorosea*, *C. militaris*, *B. bassiana*, and *N. crassa*. It was found that the homologous genes in these other species all consist of four exons (Fig. 1B). Compared to its closely related species, the *C. cicadae CcMAK2* gene may have undergone unique structural variation during evolution. However, similar to its fungal homologs, the core functional domain of *CcMAK2* is a single protein kinase catalytic domain. The putative fourth exon does not encode an independent domain but, together with exons 1, 2, and 3, contributes to encoding the protein kinase domain. Therefore, *CcMAK2* does not represent a case of “functional loss”; rather, it achieves an equivalent function through a simplified gene structure. Furthermore, the genomic region harboring *CcMAK2* was analyzed. In *C. cicadae*, *CcMAK2* is located within a highly conserved gene cluster: it is immediately upstream of a gene encoding a Sec23/Sec24 protein and downstream of genes encoding SGT1 and RecF/RecN/SMC proteins. This distinctive gene arrangement, including both the order and transcriptional orientation of the genes, is maintained with nearly perfect conservation in *C. fumosorosea*, *C. militaris*, *B. bassiana*, and *N. crassa* (Fig. 1C).

Phylogenetic analysis indicated that CcMAK2 shares a close evolutionary relationship with MAK2 homologous proteins from other representative cordycipitoid fungi and previously reported ascomycete species (Fig. 1D). It exhibits the closest affinity to *C. fumosorosea* with 89% homology, and 88% homology to *N. crassa*. Prediction of conserved domains revealed that the typical domain architecture found in these fungal MAK2 homologs is also conserved containing a protein kinase domain.

### Construction of *CcMAK2* overexpression and knockout strains

**Figure 2.**
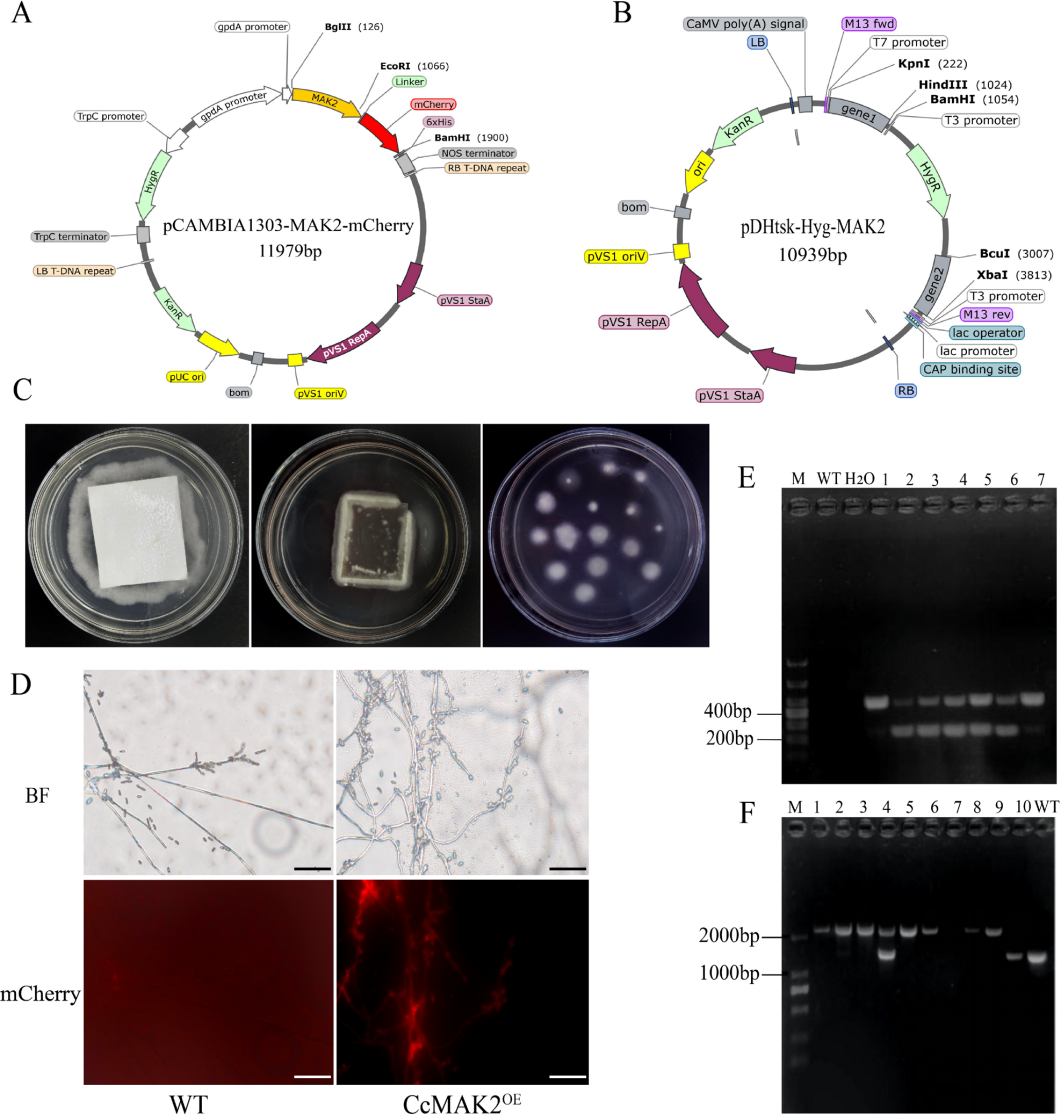
Construction of *CcMAK2* overexpression and knockout strains in *C. cicadae*. **A** Schematic diagram of the *CcMAK2* overexpression vector; **B** Schematic diagram of the *CcMAK2* knockout vector; **C***A. tumefaciens*-mediated genetic transformation of *C. cicadae* and screening of positive transformants; **D** Fluorescence detection of the overexpression strain CcMAK2^OE^ (Scale bar: 50 μm); **E** PCR verification of the overexpression strain CcMAK2^OE^ for the *Hyg* gene (468 bp) and the *mCherry* gene (229 bp); **F** PCR verification of the knockout mutant ΔCcMAK2, showing the target band of 2208 bp. M denotes the DNA molecular weight marker. BF: Bright field.

In order to investigate the function of *CcMAK2*, gene overexpression and knockout constructs were generated (Fig. 2A, B) and introduced into the WT strain via *A. tumefaciens*-mediated transformation (ATMT) (Fig. 2C). PCR analysis indicated that five putative CcMAK2^OE^ transformants yielded amplification products for the hygromycin resistance (Hyg) gene (468 bp) and the *mCherry* gene (229 bp) (Fig. 2E), consistent with the expected results and confirming the successful integration of the *CcMAK2* overexpression cassette into the WT genome. For the putative ΔCcMAK2 transformants, transformant 4^#^ produced two bands, suggesting incomplete homologous recombination; 7^#^ showed no amplification; 10^#^ exhibited the same band pattern as the WT; while the remaining seven transformants yielded only a single target fragment of 2208 bp, which differed from the 1358 bp fragment amplified from the WT strain (Fig. 2F). This pattern aligns with the expected knockout outcome, confirming that homologous recombination had replaced the *CcMAK2* locus with the hygromycin resistance cassette in these seven transformants. Fluorescence microscopy observation confirmed the expression of *mCherry* in the *C. cicadae* overexpression strains (Fig. 2D), providing further evidence for the successful integration and expression of the *CcMAK2* gene in the WT background. In total, seven *CcMAK2* knockout transformants and five *CcMAK2* overexpression transformants were obtained. For all subsequent quantitative phenotypic analyses, three randomly selected and verified independent transformants from each group were used as biological replicates, and the results are presented as the mean ± SD.

### Effect of the *CcMAK2* gene on colony growth and sporulation

**Figure 3.**
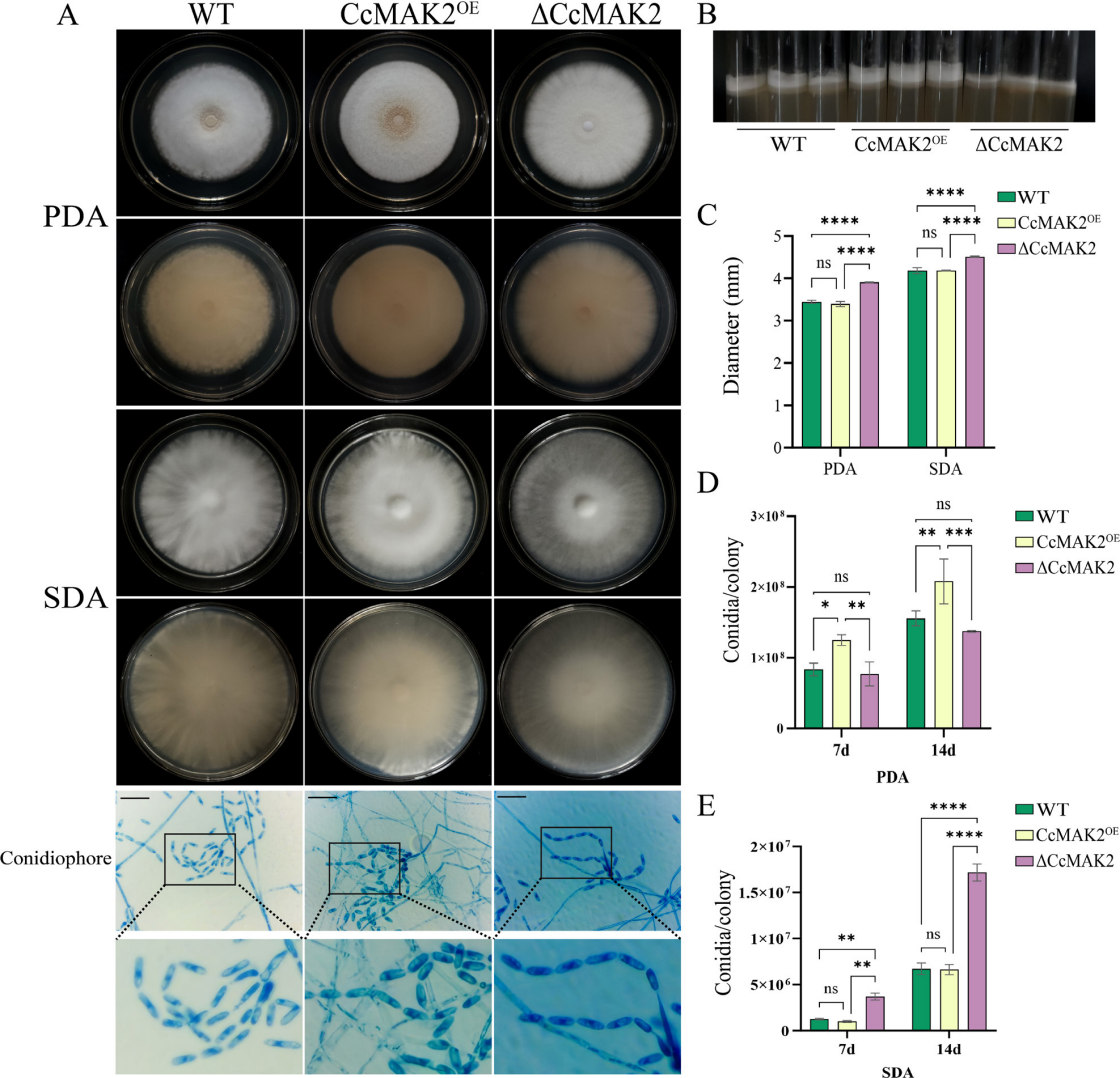
Effects of *CcMAK2* on morphology and sporulation in *C. cicadae*. **A** Colony and conidial morphology of the three different strains grown on PDA and SDA media for 14 days (scale bar: 50 μm); **B** Aerial mycelia from cultures grown in SDA slants for 7 days; **C** Colony diameters of the different strains; **D** Conidial yields of the different strains on PDA medium; **E** Conidial yields of the different strains on SDA medium. Error bars represent the SD of three biological replicates. Asterisks denote statistical significance: *p < 0.05, **p < 0.01, ***p < 0.001, ****p < 0.0001. Data are derived from measurements of three independent knockout transformants and three independent overexpression transformants (n = 3).

The WT, CcMAK2^OE^, and ΔCcMAK2 strains were cultured on PDA medium for 14 days. Both the WT and CcMAK2^OE^ colonies exhibited a yellow pigmentation at the center with dense and short mycelia, showing no significant morphological difference between them. In contrast, the ΔCcMAK2 colony lacked central pigmentation and displayed sparse mycelium. Microscopic observation revealed no apparent difference in conidial morphology among the three strains, with all producing long-oval conidia (Fig. 3A). When cultured on SDA medium for 14 days, the WT and CcMAK2^OE^ strains formed colonies with thicker mycelia at the center, whereas the ΔCcMAK2 strain produced sparse mycelia. In SDA slants, the CcMAK2^OE^ strain developed the most abundant aerial mycelium, while the ΔCcMAK2 strain produced the least (Fig. 3B). On both media, the colony diameter of the ΔCcMAK2 strain was significantly larger than those of the WT and CcMAK2^OE^ strains (p < 0.0001) (Fig. 3C). These results indicate that *CcMAK2* influences mycelial growth rate and aerial hyphal development.

A further comparison of the sporulation capacity among the different strains revealed that, on PDA medium after 7 and 14 days of cultivation, the sporulation yield of the CcMAK2^OE^ strain was significantly higher than that of the WT and ΔCcMAK2 strains (Fig. 3D). However, when cultivated on SDA medium for 7 and 14 days, the ΔCcMAK2 strain produced a significantly higher sporulation yield compared to the WT and CcMAK2^OE^ strains (Fig. 3E). These findings indicate that the *CcMAK2* gene influences asexual sporulation, but the direction of its regulation is dependent on the nutritional conditions.

### Effect of *CcMAK2* on fruiting body development

**Figure 4.**
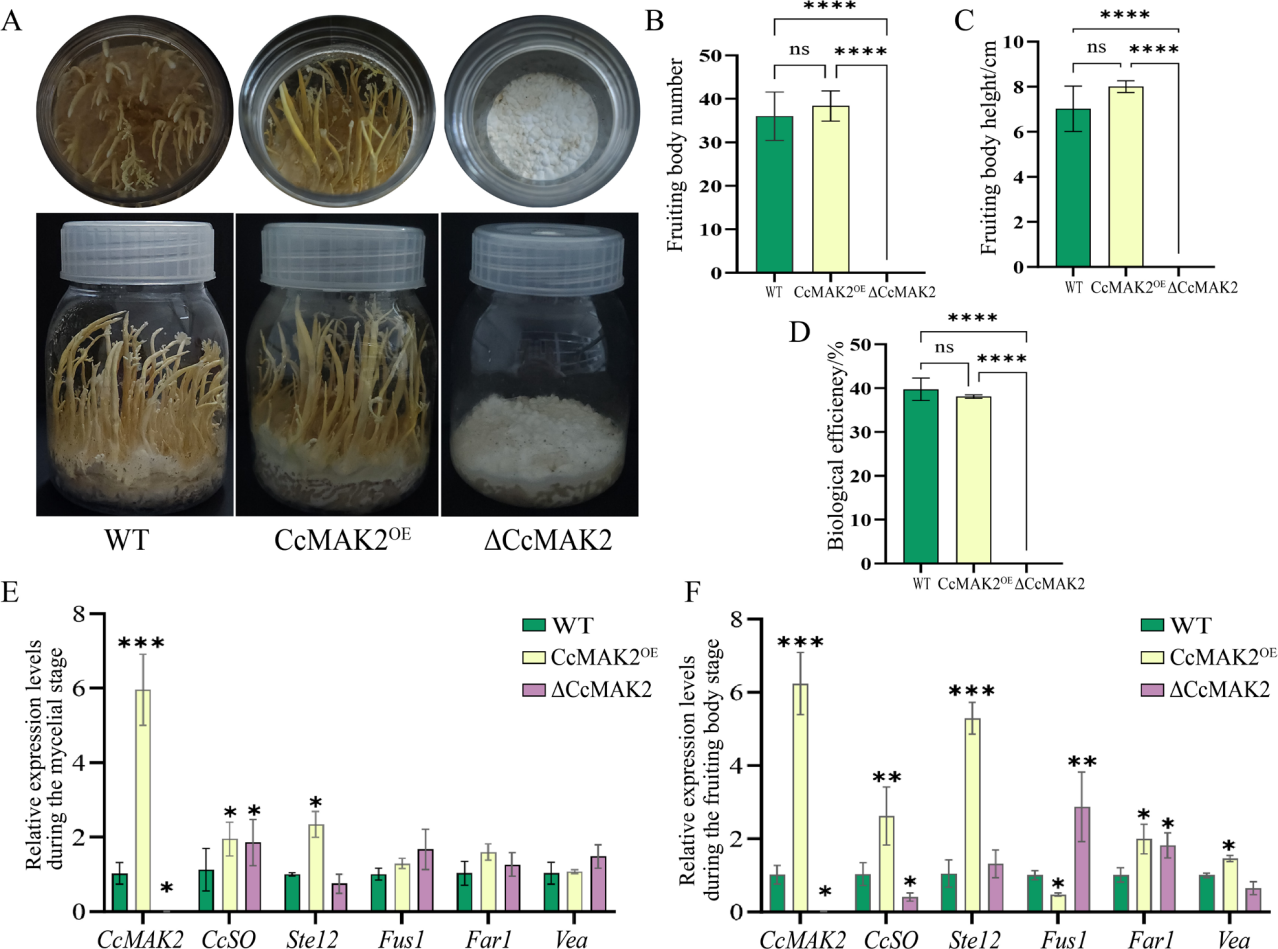
Effect of *CcMAK2* on fruiting body formation in *C. cicadae*. **A** Artificial cultivation of fruiting bodies by different strains; **B** Number of fruiting bodies produced by different strains; **C** Length of fruiting bodies from different strains; **D** Biological efficiency of fruiting body formation for different strains; **E** RT-qPCR analysis of gene expression levels in different strains during the mycelial phase; **F** RT-qPCR analysis of gene expression levels in different strains during the fruiting body formation stage. Error bars represent the SD of three biological replicates. Asterisks denote statistical significance: *p < 0.05, **p < 0.01, ****p < 0.0001. Data are derived from measurements of three independent knockout transformants and three independent overexpression transformants (n = 3).

The WT, CcMAK2^OE^, and ΔCcMAK2 strains were cultivated on rice medium for fruiting body production. Both the WT and CcMAK2^OE^ strains produced normal fruiting bodies, showing no significant differences in fruiting body number, height, or biological efficiency (Fig. 4B–D). In contrast, the ΔCcMAK2 strain lost the ability to form fruiting bodies and remained in a white mycelial state (Fig. 4A). RT-qPCR analysis of the transcript levels of MAPK pathway-related genes revealed that during the mycelial phase, the expression of *CcMAK2*, *CcSO*, and *Ste12* was significantly up-regulated in the CcMAK2^OE^ strain, while only *CcMAK2* was significantly down-regulated in the ΔCcMAK2 strain. The expression of other target genes showed no marked changes among the three strains (Fig. 4E). This expression pattern changed significantly during the fruiting body development stage. In the CcMAK2^OE^ strain, the transcript levels of *CcMAK*2, *CcSO*, *Ste12*, and *Vea* were significantly up-regulated, whereas that of *Fus1* was significantly down-regulated. In the ΔCcMAK2 strain, only *CcMAK2* and *CcSO* were significantly down-regulated, *Fus1* was significantly up-regulated, and *Far1* was significantly up-regulated in both the CcMAK2^OE^ and ΔCcMAK2 strains. These results demonstrate that *CcMAK2* is a pivotal gene for the transition from vegetative growth to reproductive growth in *C. cicadae*. The complete loss of fruiting body formation ability in the ΔCcMAK2 mutant confirms that *CcMAK2* is essential for fruiting body initiation.

### Effect of *CcMAK2* on the virulence of *C. cicadae* in insect infection

**Figure 5.**
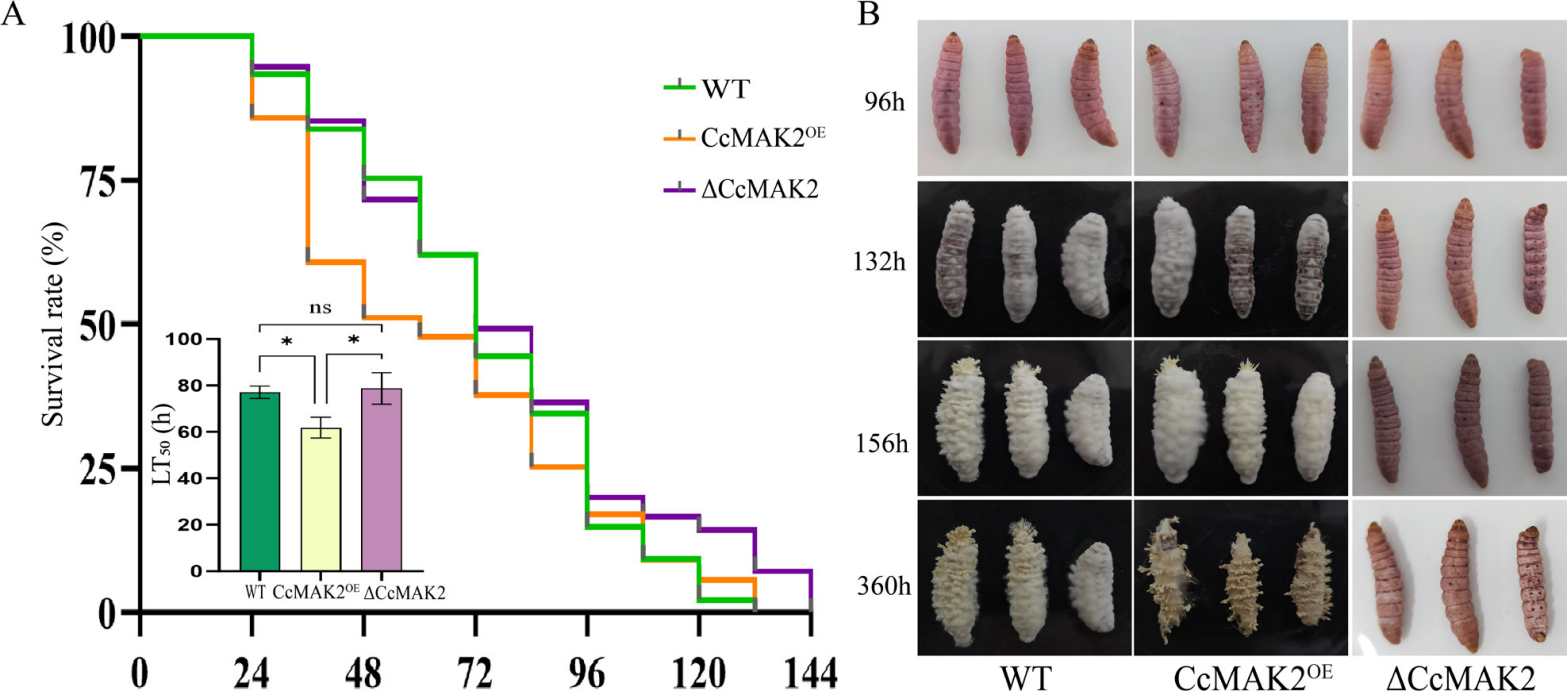
Bioassay of *CcMAK2* function during insect infection. **A** Survival rates of host insects following cuticle inoculation with the three strains; **B** Process of hyphal emergence through the insect cuticle after host death. Error bars represent the SD of three biological replicates. Asterisks denote statistical significance: *p < 0.05. Data are derived from measurements of three independent knockout transformants and three independent overexpression transformants (n = 3).

To further investigate the role of *CcMAK2* in host infection, a bioassay was conducted using greater wax moth (*G. mellonella*) larvae as the host insect via cuticle inoculation. The results showed that the median lethal times (LT_50_) for the WT, CcMAK2^OE^, and ΔCcMAK2 strains were 74 ± 3 h, 64 ± 4 h, and 78 ± 3 h, respectively. The LT_50_ of the CcMAK2^OE^ strain was significantly different from those of both the WT and ΔCcMAK2 strains (p < 0.05), whereas no significant difference was observed between the WT and ΔCcMAK2 strains (Fig. 5A). Cadavers collected at the same time point and incubated under high humidity revealed that the ΔCcMAK2 strain lost the ability to emerge through the insect cuticle (Fig. 5B). These findings indicate that overexpression of *CcMAK2* enhances fungal virulence, leading to faster host mortality. Although deletion of *CcMAK2* did not significantly affect the speed of kill, it abolished the ability of hyphae to penetrate the cuticle from insect inside and form reproductive structures on the host surface.

### Role of *CcMAK2* in redox homeostasis and cell wall development

**Figure 6.**
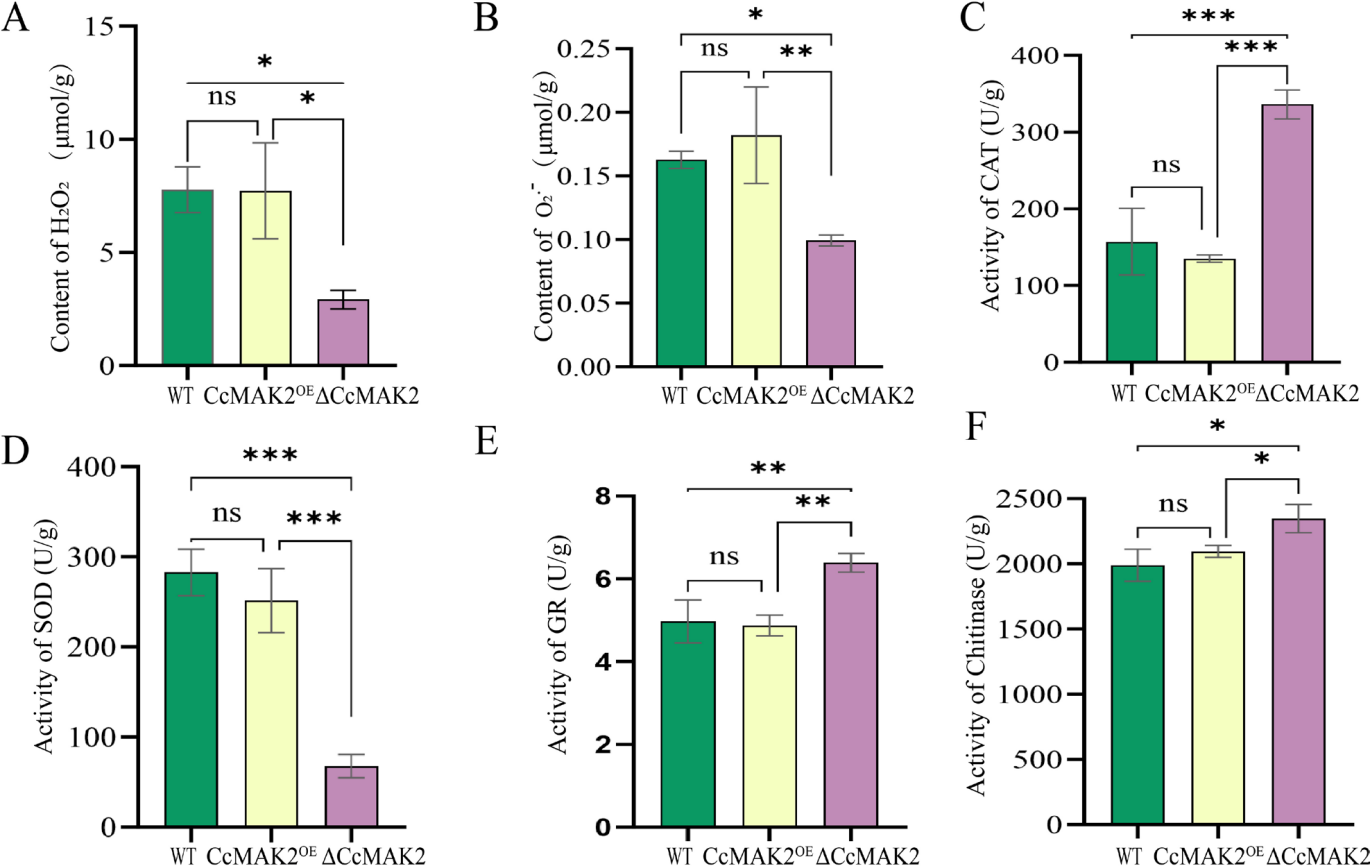
Reactive oxygen species (ROS) levels and chitinase activity in *C. cicadae*. **A** Hydrogen peroxide (H_2_O_2_) content in different strains; **B** Superoxide anion (O_2_**^.^**^-^) content in different strains; **C** Catalase (CAT) activity in different strains; **D** Superoxide dismutase (SOD) activity in different strains; **E** Glutathione reductase (GR) activity in different strains; **F** Chitinase activity in different strains. Error bars represent the SD of three biological replicates. Asterisks denote statistical significance: *p < 0.05, **p < 0.01, ***p < 0.001. Data are derived from measurements of three independent knockout transformants and three independent overexpression transformants (n = 3).

ROS can act as second messengers to activate the MAPK cascade, influencing vegetative growth and the pathogenic process in pathogenic fungi (Ray et al. 2012; Marschall and Tudzynski 2016). To investigate the impact of *CcMAK2* on ROS metabolism in the fruiting bodies of *C. cicadae*, the contents of H_2_O_2_ and O_2_**^.^**–, as well as the activities of CAT, SOD, and GR, were measured in different strains during the fruiting body stage. The results revealed no significant changes in H_2_O_2_ and O_2_^.^– contents between the WT and CcMAK2^OE^ strains, whereas these levels were significantly decreased in the ΔCcMAK2 strain compared to both (Fig. 6A, B). Concomitantly, the total activities of several antioxidant enzymes were altered: CAT and GR activities were significantly upregulated, while SOD activity was significantly downregulated in the ΔCcMAK2 strain, with no significant changes observed in the WT and CcMAK2^OE^ strains (Fig. 6C–E). This indicates that *CcMAK2* is required for maintaining appropriate ROS levels during fruiting body development. Deletion of *CcMAK2* leads to aberrant ROS levels (significant decreases in H_2_O_2_ and O_2_^.^–) and dysregulation of antioxidant enzyme activities, which is directly correlated with its defective fruiting body development phenotype. This suggests that *CcMAK2* influences the proper transmission of developmental signals by regulating ROS homeostasis.

The aforementioned results indicate that *CcMAK2* positively affects the expression of *CcSO*, a gene associated with the cell wall integrity pathway. Moreover, the ΔCcMAK2 strain exhibited severe developmental defects, remaining in a mycelial state and failing to form primordia. Therefore, measuring chitinase activity can help us more comprehensively reveal the overall changes in cell wall-related parameters under the condition of *CcMAK2* deletion. The assay revealed that chitinase activity in the ΔCcMAK2 strain was significantly higher than in the WT and CcMAK2^OE^ strains (p < 0.05), while no significant difference was observed between the WT and CcMAK2^OE^ strains (Fig. 6F). These findings suggest that *CcMAK2* likely affects normal fruiting body development by influencing the metabolic factors related to the cell wall.

## Discussion

Fungal fruiting body formation is a complex developmental process involving the coordinated action of numerous genes and their products, which may entail the activation of a cell wall stress-dependent MAPK pathway at the maturation stage, potentially playing a key role in morphological development (Wang et al. 2020). This study identified *CcMAK2* in *C. cicadae* as a homolog of *N. crassa MAK2*. It is an ortholog of yeast Fus3-MAPK and constitutes a core component of the PR pathway within the MAPK signaling cascade. The research demonstrates that *CcMAK2* is essential for the initiation and establishment of fruiting bodies in *C. cicadae*, and performs multiple functions in regulating ROS homeostasis, cell wall metabolism, and pathogenicity.

Collinearity analysis of the genomic region harboring *CcMAK2* in *C. cicadae* revealed that this gene resides within a highly conserved gene cluster, with its genomic order and orientation being nearly identical across multiple fungal species, including *C. fumosorosea*, *C. militaris*, *B. bassiana*, and *N. crassa*. The maintenance of synteny among orthologous genes across different species is generally considered a strong genomic signal, suggesting potential functional interrelationships among these genes, a linkage that has likely been maintained by evolutionary selective pressure (Yanai et al. 2002; Marcet-Houben and Gabaldón 2019). Sec23 and Sec24 are components of the COPII vesicle coat proteins, involved in protein transport from the endoplasmic reticulum (ER) to the Golgi apparatus (Jeon et al. 2014). MAK2 is a key kinase within the MAPK signaling cascade, a pathway ubiquitous in fungi that regulates diverse biological processes including cell differentiation, growth, development, stress response, and virulence (Leeder et al. 2013; Frawley and Bayram 2020; Lv et al. 2023; Xie et al. 2023; Vandermeulen et al. 2024). In fungi, SGT1 may be involved in proteostasis, cell cycle regulation, or stress response, and may engage in crosstalk with the MAPK pathway (Austin et al. 2002). RecF and RecN are proteins involved in DNA repair, specifically functioning in the recombinational repair pathway, while SMC (Structural Maintenance of Chromosomes) proteins are closely associated with chromosome structure maintenance, DNA replication, and repair (Henrikus et al. 2019; Kim et al. 2023). Thus, the finding that *CcMAK2* is tightly linked with Sec23/Sec24, SGT1, and RecF/RecN/SMC genes within a highly conserved cluster suggests significant functional conservation and potential synergistic interactions among them in fungi, possibly involving collective participation in core biological processes such as MAPK signaling, protein trafficking, proteostasis, and genome maintenance (Wizaza et al. 2018; Marcet-Houben and Gabaldón 2019).

The yeast Fus3/Kss1 pathway is relatively well characterized. Upon binding of pheromone peptides to pheromone receptors of the opposite mating type, the G protein α subunit is activated by GTP binding and dissociates from the βγ dimer. This activates downstream effectors such as Ste20, which then amplifies the signal through the Ste11-Ste7-Fus3/Kss1 (MAP3K-MAPKK-MAPK) kinase cascade in response to pheromone. Downstream, the cell cycle-dependent kinase inhibitor Far1 and the Fus3-targeted transcription factor Ste12 respond to this signal. They regulate other genes, such as *Fus1*, to promote the fusion of haploid cells of opposite mating types into diploids, thereby initiating the sexual reproductive cycle (Good et al. 2009; Bayram et al. 2012; Muller et al. 2016). This study found that deletion of *CcMAK2*, a homolog of Fus3/MAK2, completely abrogated the ability of *C. cicadae* to form fruiting bodies, arresting development at the vegetative mycelial stage. This phenotype indicates that *CcMAK2* acts as an essential “molecular switch” required for initiating the transition from vegetative growth to reproductive growth in *C. cicadae*. This finding highlights the high conservation of the core function of Fus3/MAK2, reported in regulating sexual development across species, including yeast, *Aspergillus nidulans*, and *N. crassa* (Breitkreutz et al. 2001; Li et al. 2005; Bayram et al. 2012). In contrast to the deletion phenotype, overexpression of *CcMAK2* (CcMAK2^OE^) did not confer a significant gain of function in fruiting body number, morphology, or biological efficiency. This “overexpression-no-phenotype” phenomenon contradicts the common expectation in many other systems where augmenting a signaling pathway often enhances development. It suggests that the function of *CcMAK2* may require cooperation with specific substrates or co-activators, which might already be limiting factors under wild-type conditions. Our RT-qPCR data showed that during the fruiting body stage, the expression of *Ste12* and *Vea* was significantly upregulated in the CcMAK2^OE^ strain, implying that *CcMAK2* may indeed enhance the activity of the nuclear transcriptional regulatory module, consistent with reports by Bayram et al. (2012) and Atoui et al. (2008). However, the key downstream effector gene, *Fus1*, was downregulated. This may suggest that *CcMAK2* operates through a complex regulatory network, simultaneously activating pro-developmental factors (e.g., Ste12-Vea) and repressing specific branches (e.g., Fus1, involved in membrane fusion) to balance the developmental process. It is speculated that the low expression of *Fus1* is due to the lack of cells with the opposite mating type, which hinders the formation of diploids and the initiation of sexual reproduction.

Furthermore, the effect of *CcMAK2* on conidiation exhibited opposite patterns depending on the culture medium. On PDA medium, the CcMAK2^OE^ strain showed the highest conidiation level, aligning with reports of this pathway positively regulating asexual sporulation in fungi such as *B. bassiana* (Zhao et al. 2023). However, on SDA medium, the ΔCcMAK2 mutant exhibited the highest conidiation level. This nutrient-dependent phenomenon likely reflects the differences in nutritional components between the two media. PDA is the most commonly used basal medium in mycology. Its composition has a relatively high carbon-to-nitrogen (C/N) ratio, including natural complex carbon and nitrogen sources, phytohormone, vitamins, trace elements and glucose (as the primary carbon source), etc. SDA is another widely used fungal medium, composed of glucose (carbon source) and peptone (nitrogen source). Compared to PDA, SDA has a more defined and abundant nitrogen source resulting in a lower C/N ratio. The nutritional differences likely serve as critical environmental signals leading to the markedly opposite conidiation phenotypes exhibited by the different *CcMAK2* strains on each medium. Under different environmental signals, the fungus may induce distinct signaling pathway to regulate conidiation. Based on the current data, we hypothesize that the fungus senses the abundance and type of nitrogen sources and other important components in the environment through an as-yet-unidentified nutrient-sensing system. These signals may be transduced via distinct pathways. Under PDA conditions (relatively nitrogen-limited and complex in composition), MAK2 acts as a positive regulatory factor, and the signal is transmitted through the canonical Ste11-Ste7-Fus3/MAK2 MAPK cascade. This cascade activates downstream transcription factors, which in turn promote the expression of conidiation-related genes. In contrast, under SDA conditions (nitrogen-rich and single carbon source), the simple nutritional signal may simultaneously activate multiple signaling pathways. Upon *CcMAK2* deletion, these alternative pathways may be upregulated to compensate for its function. Alternatively, under nitrogen-rich conditions, *CcMAK2* might interact with distinct co-factors to repress certain conidiation-promoting genes. This hypothesis warrants future investigation to validate.

ROS are continuously generated during normal fungal metabolism, and their accumulation can lead to cytotoxic effects such as protein carbonylation, lipid peroxidation, and DNA oxidative damage, even triggering programmed cell death. However, at appropriate concentrations, ROS can act as secondary messengers to activate the MAPK cascade, influencing vegetative growth and the pathogenic processes of phytopathogenic fungi (Ray et al. 2012; Marschall and Tudzynski 2016). This study found that, compared with the WT strain, the levels of H_2_O_2_ and O_2_^.^– in the ΔCcMAK2 mutant decreased significantly by approximately 62% and 39%, respectively, while the activities of antioxidant enzymes (CAT, GR) increased. In contrast, the CcMAK2^OE^ showed no significant changes in these parameters. These results indicate that *CcMAK2* likely functions as an upstream regulator maintaining appropriate ROS homeostasis during development. Its deletion leads to insufficient ROS signaling, thereby failing to activate downstream developmental programs, which is consistent with the phenotype of the knockout mutant being unable to form fruiting bodies. As signaling molecules, ROS can regulate the activity of MAPK pathways. In *Pleurotus ostreatus*, mechanical damage can rapidly induce a ROS burst and activate the MAPK pathway, forming the core of the early defense response (Zhu et al. 2025).

In the pathogenicity assay, the ΔCcMAK2 mutant showed no significant impact on the median lethal time (LT_50_) against *G. mellonella*, but it lost the ability to penetrate the cuticle from insect inside. This indicates that *CcMAK2* is crucial for completing the infection cycle (penetration of the host and external proliferation). This finding is largely consistent with the report that a *B. bassiana* mutant lacking the Fus3/Kss1 MAPK homolog (*Bbmpk1*) completely lost both cuticle penetration ability and pathogenicity in bioassays (Zhao et al. 2023). However, it appears to have little effect on the initial process of rapidly killing the insect within the hemocoel.

Concurrently, the ΔCcMAK2 mutant exhibited elevated chitinase activity, and abnormal phenotypes—failure to form primordia, sparse hyphae, and loss of penetration ability. RT-qPCR data showed that deletion of *CcMAK2* led to altered transcript levels of *CcSO*, a key gene associated with the CWI pathway. These findings suggest a potential link between *CcMAK2* and the regulation of cell wall-related parameters. However, whether these changes represent direct regulation by *CcMAK2* or secondary consequences of the mutant’s severely impaired growth remains to be determined. During the developmental transition phase, this homeostasis is crucial for hyphal aggregation and differentiation into the compact tissue structure of the primordium. Our previous study demonstrated that *CcSO* is a key protein in the cell wall integrity pathway and likely plays a critical regulatory role in the development of asexual fruiting bodies in *C. cicadae* by modulating ROS homeostasis and maintaining cell wall integrity (Chen et al. 2025). This finding is analogous to reports in the apple canker pathogen *Valsa mali*, where the Fus3/Kss1-related MAPK gene *VmPmk1* regulates virulence and the expression of cell wall-degrading enzymes, playing a significant role in growth, asexual development, oxidative stress response, and the maintenance of cell wall integrity (Wu et al. 2017). The regulatory mechanisms governing gene expression are highly complex. Furthermore, the crosstalk between different signaling pathways requires further investigation.

**Figure 7.**
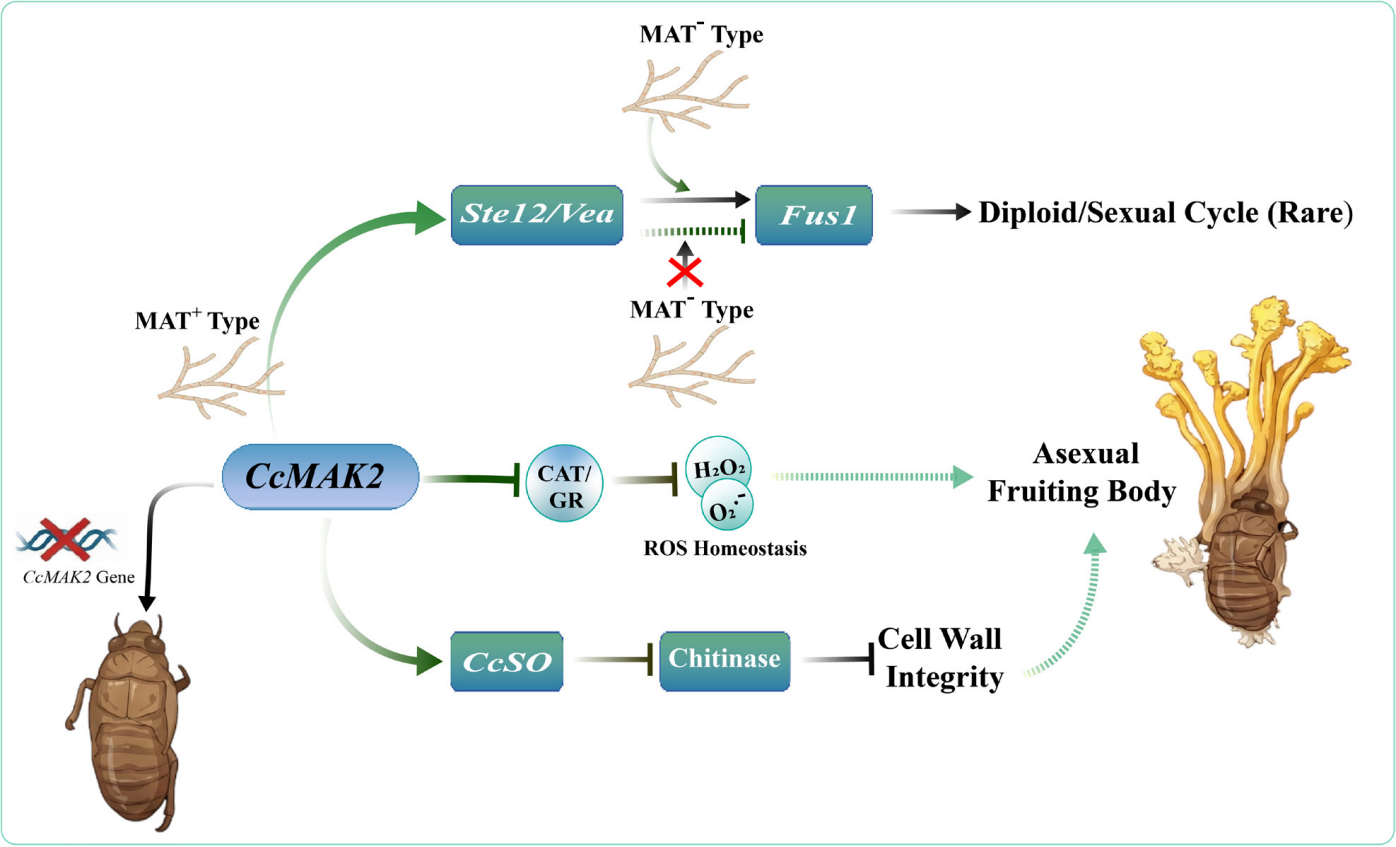
A proposed model for the role of *CcMAK2* in regulating fruiting body formation in *C. cicadae*. MAT: Mating Type; CAT: catalase; GR: glutathione reductase; ROS: reactive oxygen species. The arrow indicates positive regulation, and the solid T-shaped arrow indicates negative regulation.

Based on the above analysis, we propose a working model for *CcMAK2*-mediated regulation of fruiting body development (Fig. 7), outlining the potential roles of *CcMAK2* in these processes and providing a conceptual framework for future mechanistic studies.

## Conclusion

This study systematically elucidated the core function of *CcMAK2* in the asexual fruiting body formation of the medicinal fungus *C. cicadae*. Knockout of the *CcMAK2* gene resulted in reduced accumulation of H_2_O_2_ and O_2_^.^–, consequently leading to a complete failure in asexual fruiting body development. In contrast, both the wild-type and overexpression strains developed normally. It may act as an upstream signal to sustain the ROS homeostasis essential for developmental progression. Additionally, the observed alterations in *CcSO* transcript levels, chitinase activity and abnormal phenotypes in the ΔCcMAK2 mutant suggest that *CcMAK2* is associated with the maintenance of cell wall integrity-related parameters during development. These findings demonstrate that the *CcMAK2* gene plays a pivotal role in the morphogenesis of *C. cicadae* and constitutes an essential regulatory hub for fruiting body development.

## Additional information

### Artificial Intelligence (AI) use

The authors accept full responsibility for the content of the manuscript, including the disclosure of any use of AI.

No AI tools were used in the preparation of this manuscript.

### Adherence to national and international regulations

All the fungal strains used in this study have been legally obtained, respecting the Convention on Biological Diversity (Rio Convention).

### Author ORCIDs

Lanya Chen https://orcid.org/0009-0007-6112-2385

Xiaoli Tian https://orcid.org/0009-0008-9659-2157

Jie Han https://orcid.org/0000-0002-5682-9753

Jiehong Zhao https://orcid.org/0000-0003-2972-382X

### Data availability

All of the data that support the findings of this study are available in the main text.
